# Introducing the 2023 HRS/APHRS/LAHRS guideline on cardiac physiologic pacing for the avoidance and mitigation of heart failure: Are we entering a new age in pacing?

**DOI:** 10.1016/j.hroo.2023.08.002

**Published:** 2023-08-28

**Authors:** Julia H. Indik

**Affiliations:** Department of Medicine, University of Arizona College of Medicine, Tucson, Arizona

Until several years ago, there had been one method to provide cardiac physiologic pacing (CPP), defined as pacing that restores or preserves ventricular synchrony, that by pacing the left ventricular epicardial wall. Cardiac resynchronization therapy (CRT) has been studied in many large randomized controlled trials and with long-established guideline recommendations. Conduction system pacing (CSP), engaging the His bundle or left bundle branch fibers, is a relatively new means to achieve physiologic pacing. Cardiac physiologic pacing can be achieved either by CSP or CRT.

In the last few years, research into CSP, especially left bundle branch area pacing (LBBAP), has greatly expanded, driven primarily by observational studies^1^, ^2^, ^3^, ^4^, ^5^, ^6^, ^7^, ^8^, ^9^ and small randomized controlled trials.^10^, ^11^, ^12^ These investigations have examined a broad range of ejection fraction (EF), QRS duration, and anticipated ventricular pacing percentage. LBBAP has been shown to be feasible and with durable low pacing thresholds.^4^^,^^5^ Patients who are indicated for CRT appear to have a better response in EF and reduced heart failure hospitalization with LBBAP or His bundle pacing compared with biventricular pacing.^2^^,^^8^^,^^13^ Furthermore, one study showed that LVEF improved in heart failure patients with right bundle branch block treated with LBBAP.^14^ Patients with a right ventricular (RV) pacing–induced cardiomyopathy also benefit if upgraded to CSP.^7^ Finally, patients with a normal LVEF, but who are anticipated to need substantial ventricular pacing, do better with CSP over RV apical pacing, to maintain LVEF and reduce heart failure hospitalization and need for biventricular pacing upgrade.^3^^,^^15^ These studies have substantially increased our understanding of the feasibility of CSP and indicate that a large range of patients are likely to benefit from this pacing technique.

The publication of the 2023 Heart Rhythm Society/Asia Pacific Heart Rhythm Society/Latin American Heart Rhythm Society Guideline on Cardiac Physiologic Pacing for the Avoidance and Mitigation of Heart Failure^16^ brings together the large body of CRT research and the newer studies for CSP to provide new recommendations (Table 1). For patients with an ejection fraction (EF) below 35% and New York Heart Association (NYHA) functional class II to IV symptoms on guideline-directed medical therapy (GDMT), recommendations for CRT continue as previously published.^17^ There is a new class I CRT recommendation for female patients with QRS durations of 120 to 149 ms, as substudy analyses of the Multicenter Automatic Defibrillator Implantation Trial with Cardiac Resynchronization Therapy^18^ and Resynchronization-Defibrillation for Ambulatory Heart Failure Trial^19^ showed improved survival and fewer heart failure events compared with men. Also, CSP is broadly recommended as a bailout-type strategy whenever CRT cannot be achieved.

**Table 1:** Key new recommendations for physiologic pacing

Patient population	Recommendation	Class
Female	In patients with select characteristics (eg, female sex) who have LVEF ≤35%, sinus rhythm, LBBB with QRS duration 120–149 ms, and NYHA functional class II–IV symptoms on GDMT, CRT with BiV pacing is recommended to reduce mortality and HF events and to improve LVEF.	1
CSP as bailout if CS LV lead unsuccessful	In patients undergoing CRT with BiV pacing implantation via the CS, crossover to CSP with HBP or LBBAP is reasonable when the CS LV lead placement is unsuccessful or suboptimal.	2a
LBBB, LV systolic dysfunction	In patients with LVEF 36%–50%, sinus rhythm, LBBB with QRS duration ≥150 ms, and NYHA functional class II–IV symptoms on GDMT, CPP may be considered to maintain or improve LVEF.	2b
	In patients with LVEF ≤35%, sinus rhythm, LBBB with a QRS duration ≥150 ms, and NYHA functional class II–IV symptoms on GDMT, CSP with HBP or LBBAP may be considered as an alternative to CRT with BiV pacing.	2b
PICM	In patients with a CIED with a decline in LV function or worsening of HF symptoms attributed to substantial ventricular pacing, CRT with BiV pacing is recommended to improve LV function and improve HF symptoms.	1
	In patients with a CIED with a decline in LV function or worsening of HF symptoms attributed to substantial ventricular pacing, revision of CIED to a CSP device can be beneficial to improve LV function and symptoms of HF.	2a
Anticipated substantial RVP, normal LVEF	In patients with normal LVEF who are anticipated to require substantial ventricular pacing, it may be reasonable to treat patients with CPP to reduce the risk of PICM.	2b
Anticipated less than substantial RVP, normal LVEF	In patients who are undergoing permanent pacing with normal LVEF and are anticipated to require less than substantial ventricular pacing, an LBBAP lead may be considered as an alternative to an RVP lead.	2b
	In patients with normal LVEF who are anticipated to require less than substantial ventricular pacing, CRT with BiV pacing is not indicated.	3: No benefit

BiV = biventricular; CS = coronary sinus; CIED = cardiovascular implantable electrical device; CPP = cardiac physiologic pacing; CRT = cardiac resynchronization therapy; CSP = conduction system pacing; GDMT = guideline-directed medical therapy; HBP = His bundle pacing; HF = heart failure; LBBAP = left bundle branch area pacing; LBBB = left bundle branch block; LV = left ventricular; LVEF = left ventricular ejection fraction; PICM = pacing-induced cardiomyopathy; RVP = right ventricular pacing.

Left bundle branch block (LBBB) itself may impair systolic function, and 2 new recommendations address this patient population. Specifically for patients with EF of 36% to 50%, in sinus rhythm with LBBB, QRS duration ≥150 ms, and NYHA functional class II to IV, CPP (CRT or CSP) is a class 2b recommendation, and for EF ≤35% CSP is a class 2b alternative to CRT. Additionally, for patients with a pacing-induced cardiomyopathy, CRT is recommended as class 1, and CSP as class 2a.

The guideline also recognizes patients who may have a normal EF but are at risk for cardiomyopathy with substantial RV pacing. In the 2018 American College of Cardiology bradycardia guideline,^20^ recommendations for His bundle pacing were made for substantial RV pacing but only in the presence of already compromised systolic function or complete heart block at the level of the atrioventricular node. The new guideline now expands this to patients with substantial RV pacing burdens with a normal LVEF offering a class 2b recommendation for CPP (CRT or CSP).

And what of patients who do not have LBBB, who are not anticipated to have substantial ventricular pacing, and who have a normal EF? Given the large body of evidence that has shown LBBAP to provide effective pacing with durable low pacing thresholds, a class 2b recommendation is now given to this patient population, whereas CRT is a class 3, no benefit.

The 2023 Heart Rhythm Society/Asia Pacific Heart Rhythm Society/Latin American Heart Rhythm Society Guideline on Cardiac Physiologic Pacing for the Avoidance and Mitigation of Heart Failure is a very welcome addition to cardiac rhythm practice guidance. As research in CSP continues to accelerate, including future randomized controlled trials with long-term follow up, we may see a major shift in the approach to pacing.
